# Supplementary material for: Resolving the full spectrum of human genome variation using Linked-Reads
Source: Genome Res. 2019 Apr;29(4):635–45. doi: 10.1101/gr.234443.118 (PMC6442396; doi:10.1101/gr.234443.118)
Supplement: Supplemental Material [file supp_gr.234443.118_Supplemental_longranger-2.2.zip › longranger-2.2.tar.bz2!/lr2.2-public/lib/stan/cmdstan/stan/doc/api/html/var__decls__grammar__def_8hpp.html]

Stan: src/stan/gm/grammars/var\_decls\_grammar\_def.hpp File Reference


|  |  |
| --- | --- |
|  | Stan  2.5.0  probability, sampling & optimization |


- Main Page
- Related Pages
- Namespaces
- Classes
- Files

- File List
- File Members

All Classes Namespaces Files Functions Variables Typedefs Enumerations Enumerator Friends Macros Pages

- src
- stan
- gm
- grammars

Classes |
Functions |
Variables

var\_decls\_grammar\_def.hpp File Reference

`#include <boost/spirit/include/qi.hpp>`  
`#include <boost/spirit/include/phoenix_core.hpp>`  
`#include <boost/spirit/include/phoenix_function.hpp>`  
`#include <boost/spirit/include/phoenix_fusion.hpp>`  
`#include <boost/spirit/include/phoenix_object.hpp>`  
`#include <boost/spirit/include/phoenix_operator.hpp>`  
`#include <boost/spirit/include/phoenix_stl.hpp>`  
`#include <boost/lexical_cast.hpp>`  
`#include <boost/fusion/include/adapt_struct.hpp>`  
`#include <boost/fusion/include/std_pair.hpp>`  
`#include <boost/config/warning_disable.hpp>`  
`#include <boost/spirit/include/qi_numeric.hpp>`  
`#include <stan/gm/ast.hpp>`  
`#include <stan/gm/grammars/var_decls_grammar.hpp>`  
`#include <stan/gm/grammars/common_adaptors_def.hpp>`

Go to the source code of this file.

|  |  |
| --- | --- |
| Classes | |
| struct | data\_only\_expression |
|  | |
| struct | add\_var |
|  | |
| struct | add\_var::result< T1, T2, T3, T4, T5, T6 > |
|  | |
| struct | validate\_decl\_constraints |
|  | |
| struct | validate\_decl\_constraints::result< T1, T2, T3, T4 > |
|  | |
| struct | validate\_identifier |
|  | |
| struct | validate\_identifier::result< T1, T2 > |
|  | |
| struct | copy\_square\_cholesky\_dimension\_if\_necessary |
|  | |
| struct | copy\_square\_cholesky\_dimension\_if\_necessary::result< T1 > |
|  | |
| struct | empty\_range |
|  | |
| struct | empty\_range::result< T1 > |
|  | |
| struct | validate\_int\_expr |
|  | |
| struct | validate\_int\_expr::result< T1, T2, T3 > |
|  | |
| struct | set\_int\_range\_lower |
|  | |
| struct | set\_int\_range\_lower::result< T1, T2, T3, T4 > |
|  | |
| struct | set\_int\_range\_upper |
|  | |
| struct | set\_int\_range\_upper::result< T1, T2, T3, T4 > |
|  | |
| struct | validate\_int\_data\_expr |
|  | |
| struct | validate\_int\_data\_expr::result< T1, T2, T3, T4, T5 > |
|  | |
| struct | validate\_double\_expr |
|  | |
| struct | validate\_double\_expr::result< T1, T2 > |
|  | |
| struct | set\_double\_range\_lower |
|  | |
| struct | set\_double\_range\_lower::result< T1, T2, T3, T4 > |
|  | |
| struct | set\_double\_range\_upper |
|  | |
| struct | set\_double\_range\_upper::result< T1, T2, T3, T4 > |
|  | |

|  |  |
| --- | --- |
| Functions | |
|  | BOOST\_FUSION\_ADAPT\_STRUCT (stan::gm::int\_var\_decl,(stan::gm::range, range\_)(std::string, name\_)(std::vector< stan::gm::expression >, dims\_)) BOOST\_FUSION\_ADAPT\_STRUCT(stan |
|  | |

|  |  |
| --- | --- |
| Variables | |
| boost::phoenix::function< add\_var > | add\_var\_f |
|  | |
| boost::phoenix::function  < validate\_decl\_constraints > | validate\_decl\_constraints\_f |
|  | |
| boost::phoenix::function  < validate\_identifier > | validate\_identifier\_f |
|  | |
| boost::phoenix::function  < copy\_square\_cholesky\_dimension\_if\_necessary > | copy\_square\_cholesky\_dimension\_if\_necessary\_f |
|  | |
| boost::phoenix::function  < empty\_range > | empty\_range\_f |
|  | |
| boost::phoenix::function  < validate\_int\_expr > | validate\_int\_expr\_f |
|  | |
| boost::phoenix::function  < set\_int\_range\_lower > | set\_int\_range\_lower\_f |
|  | |
| boost::phoenix::function  < set\_int\_range\_upper > | set\_int\_range\_upper\_f |
|  | |
| boost::phoenix::function  < validate\_int\_data\_expr > | validate\_int\_data\_expr\_f |
|  | |
| boost::phoenix::function  < validate\_double\_expr > | validate\_double\_expr\_f |
|  | |
| boost::phoenix::function  < set\_double\_range\_lower > | set\_double\_range\_lower\_f |
|  | |
| boost::phoenix::function  < set\_double\_range\_upper > | set\_double\_range\_upper\_f |
|  | |

## Function Documentation

|  |  |  |  |
| --- | --- | --- | --- |
| BOOST\_FUSION\_ADAPT\_STRUCT | ( | stan::gm::int\_var\_decl | , |
|  |  | (stan::gm::range, range\_)(std::string, name\_)(std::vector< stan::gm::expression >, dims\_) |  |
|  | ) |  |  |

Definition at line 22 of file var\_decls\_grammar\_def.hpp.

## Variable Documentation

boost::phoenix::function<add\_var> add\_var\_f

Definition at line 280 of file var\_decls\_grammar\_def.hpp.

boost::phoenix::function<copy\_square\_cholesky\_dimension\_if\_necessary> copy\_square\_cholesky\_dimension\_if\_necessary\_f

Definition at line 517 of file var\_decls\_grammar\_def.hpp.

boost::phoenix::function<empty\_range> empty\_range\_f

Definition at line 526 of file var\_decls\_grammar\_def.hpp.

boost::phoenix::function<set\_double\_range\_lower> set\_double\_range\_lower\_f

Definition at line 635 of file var\_decls\_grammar\_def.hpp.

boost::phoenix::function<set\_double\_range\_upper> set\_double\_range\_upper\_f

Definition at line 649 of file var\_decls\_grammar\_def.hpp.

boost::phoenix::function<set\_int\_range\_lower> set\_int\_range\_lower\_f

Definition at line 559 of file var\_decls\_grammar\_def.hpp.

boost::phoenix::function<set\_int\_range\_upper> set\_int\_range\_upper\_f

Definition at line 573 of file var\_decls\_grammar\_def.hpp.

boost::phoenix::function<validate\_decl\_constraints> validate\_decl\_constraints\_f

Definition at line 303 of file var\_decls\_grammar\_def.hpp.

boost::phoenix::function<validate\_double\_expr> validate\_double\_expr\_f

Definition at line 620 of file var\_decls\_grammar\_def.hpp.

boost::phoenix::function<validate\_identifier> validate\_identifier\_f

Definition at line 505 of file var\_decls\_grammar\_def.hpp.

boost::phoenix::function<validate\_int\_data\_expr> validate\_int\_data\_expr\_f

Definition at line 603 of file var\_decls\_grammar\_def.hpp.

boost::phoenix::function<validate\_int\_expr> validate\_int\_expr\_f

Definition at line 545 of file var\_decls\_grammar\_def.hpp.


---

[ Stan Home Page ]

*© 2011–2014,
Stan Development Team.*
